# Evaluation of eluforsen, a novel RNA oligonucleotide for restoration of CFTR function in *in vitro* and murine models of p.Phe508del cystic fibrosis

**DOI:** 10.1371/journal.pone.0219182

**Published:** 2019-06-28

**Authors:** Wouter Beumer, Jim Swildens, Teresinha Leal, Sabrina Noel, Herma Anthonijsz, Geert van der Horst, Hester Kuiperij-Boersma, Marko Potman, Charlotte van Putten, Patricia Biasutto, Gerard Platenburg, Hugo de Jonge, Noreen Henig, Tita Ritsema

**Affiliations:** 1 ProQR Therapeutics, Leiden, The Netherlands; 2 Université Catholique de Louvain, Louvain Centre for Toxicology and Applied Pharmacology, Brussels, Belgium; 3 Department of Gastroenterology and Hepatology, Erasmus University Medical Center, Rotterdam, The Netherlands; Emory University School of Medicine, UNITED STATES

## Abstract

Cystic fibrosis (CF) is caused by mutations in the gene encoding the epithelial chloride channel CF transmembrane conductance regulator (CFTR) protein. The most common mutation is a deletion of three nucleotides leading to the loss of phenylalanine at position 508 (p.Phe508del) in the protein. This study evaluates eluforsen, a novel, single-stranded, 33-nucleotide antisense oligonucleotide designed to restore CFTR function, in in vitro and in vivo models of p.Phe508del CF. The aims of the study were to demonstrate cellular uptake of eluforsen, and its efficacy in functional restoration of p.Phe508del-CFTR both in vitro and in vivo. In vitro, the effect of eluforsen was investigated in human CF pancreatic adenocarcinoma cells and human bronchial epithelial cells. Two mouse models were used to evaluate eluforsen in vivo. In vitro, eluforsen improved chloride efflux in CF pancreatic adenocarcinoma cell cultures and increased short-circuit current in primary human bronchial epithelial cells, both indicating restoration of CFTR function. In vivo, eluforsen was taken up by airway epithelium following oro-tracheal administration in mice, resulting in systemic exposure of eluforsen. In female F508del-CFTR mice, eluforsen significantly increased CFTR-mediated saliva secretion (used as a measure of CFTR function, equivalent to the sweat test in humans). Similarly, intranasal administration of eluforsen significantly improved nasal potential difference (NPD), and therefore CFTR conductance, in two CF mouse models. These findings indicate that eluforsen improved CFTR function in cell and animal models of p.Phe508del-CFTR-mediated CF and supported further development of eluforsen in human clinical trials, where eluforsen has also been shown to improve CFTR activity as measured by NPD.

## Introduction

Cystic fibrosis (CF) is a genetic disorder that affects the respiratory, digestive, and reproductive organs, and has a varied world-wide prevalence, with an estimated 1 in 2,000–3,000 newborns affected by CF in Europe and 1 in 3,500 newborns affected by CF in the USA [1]. Individuals with this progressive condition typically experience multisystem organ failure and ultimately premature death, with a median age at death of 27 years [2]. More specifically, CF is an autosomal recessive disease caused by mutations in the gene encoding the CF transmembrane conductance regulator (CFTR) protein [3]. CFTR is an anion channel present mainly in the apical membrane of epithelial cells, where it regulates fluid and electrolyte transport across the epithelium [4]. Therefore, a loss of CFTR function causes dehydration and acidification of the apical epithelial surface and a subsequent buildup of thick mucus, which can lead to fatal lung infections [5,6], as well as pancreatic insufficiency and poor absorption of nutrients [1].

To date, over 2,000 mutations of CFTR have been identified [7], which correspond to varying degrees of loss of CFTR function, and a spectrum of clinical presentations of CF [8]. The most common CFTR mutation is a deletion of three nucleotides in the DNA leading to the loss of a phenylalanine at position 508 (p.Phe508del) in the protein, which accounts for approximately 70% of mutated alleles in patients of European descent with CF [8,9]. The p.Phe508del mutation impairs proper biosynthesis of the CFTR protein, which is misfolded and targeted for proteosomal degradation. Furthermore, p.Phe508del-CFTR that does reach the cell surface has a reduced half-life due to increased endocytosis. Moreover, p.Phe508del-CFTR shows defects in channel gating and thermostability, and is therefore difficult to repair at the protein level [10,11].

Before the *CFTR* gene was first described in 1989 [3], CF therapies managed symptoms but did not address the underlying cause of the disease. The goal for CF therapeutic development has since shifted to restoration of functional CFTR, and to that end two main approaches have been explored to date: gene therapy and small molecules targeting the mutated protein. Gene therapy, aiming to permanently introduce wild-type (WT) CFTR, has so far demonstrated only minimal success in CF [12,13]. In contrast, several small molecules have been shown to improve CFTR protein function, as exemplified by the FDA-approved CFTR potentiator ivacaftor, which is used as monotherapy or in combination with the CFTR corrector lumacaftor [14–16]. However, while small molecule approaches have recently demonstrated robust therapeutic effects in patients with CF with specific CFTR mutations [17,18], there remains an unmet clinical need for patients with the p.Phe508del mutation.

The use of antisense oligonucleotides to modulate p.Phe508del-*CFTR* mRNA has already shown some therapeutic potential [19,20]. In 2004, Zamecnik et al. [21] demonstrated that treatment of a cultured p.Phe508del-CFTR cell line with a duplex consisting of a 33-nucleotide (nt) 2’-O-methyl-modified RNA oligonucleotide 100% homologous to WT *CFTR* mRNA, and an 11 nt RNA oligonucleotide (respectively termed CF4 and CF6), restored CFTR function [21]. Since it was not shown that both oligonucleotides in the duplex were essential for reversal of phenotype, we designed an improved version of CF4 alone, previously designated as QR-010, and now known as eluforsen (the International Non-Proprietary Name, eluforsen, is currently under review).

The aims of the present study were to demonstrate cellular uptake of eluforsen, and its efficacy in functional restoration of p.Phe508del-CFTR both in vitro and in vivo. Endpoints were selected to maximize translational relevance, and included the saliva secretion assay, which is equivalent to the sweat test endpoint used in clinical trials, and nasal potential difference (NPD), which is used to diagnose CF and is a common endpoint in clinical trials of CF therapies [16,22–24].

## Materials and methods

### Eluforsen and control oligonucleotides

Eluforsen is a 33 nt, single-stranded, fully phosphorothioated and fully 2’-O-methyl-modified oligonucleotide partly complementary to the p.Phe508del-*CFTR* RNA, and based on the CF4 molecule (5’-AUCAUAGGAAACACCAAAGAUGAUAUUUUCUUU-3’) [21]. Eluforsen was manufactured via a solid-phase synthesis followed by a chromatographic purification step, and then freeze-dried to give eluforsen as a lyophilized white to off-white solid. Phosphorothioate modifications to oligonucleotides have been previously demonstrated to protect against breakdown by nucleases and improve cellular uptake [25], therefore eluforsen was designed as a fully phosphorothioated variant of CF4. The Cy5-labeled eluforsen used in our studies contained a Cy5 label covalently bound to the 5’ end of eluforsen. A scrambled control oligonucleotide (5’-UAUUCAAGUUACACUCAAGAAGGAAUAAUUUCU-3’) with the same chemical modifications as eluforsen was used as a negative control.

For in vitro experiments, 100 μM or 1 mM stock solutions of eluforsen, the control oligonucleotide (both from Biospring, Frankfurt, Germany), and Cy5-labeled eluforsen (Axo Labs, Kulmbach, Germany) were prepared by dissolving each oligonucleotide in sterile water (Versol, Aguettant, Lyon, France), resulting in an isotonic, isoosmolar solution. For the in vivo saliva secretion assays, 5 mg/mL stock solutions (Nitto Denko Avecia, Milford, MA, USA) were prepared by dissolving eluforsen in sterile saline (0.9% NaCl in water). For the in vivo NPD experiments, 20 mg/mL stock solutions (Biospring) were prepared by dissolving eluforsen in sterile saline.

### Cell cultures

Human CF pancreatic adenocarcinoma (CFPAC-1) cells were used to assess the effect of eluforsen on CFTR function. Cells were obtained from the American Type Culture Collection (CRL-1918; Manassas, VA, USA) and cultured according to the manufacturer’s instructions [26]. To test eluforsen in CFPAC-1 cells, these cells were transfected using Lipofectamine 2000 (Life Technologies), as this results in rapid delivery of antisense oligonucleotides to these cells. Eluforsen was also tested in primary human bronchial epithelial (HBE) cells from homozygous p.Phe508del CF donors (Asterand, Royston, UK). HBE cells were seeded at a density of 1.67 × 10^4^ cells/plate in cell culture plates (Ø 100 mm, VWR, Amsterdam, the Netherlands) and cultured in keratinocyte serum-free medium supplemented with 0.2 ng/mL epidermal growth factor 1–53, 25 μg/mL bovine pituitary extract, 1% penicillin/streptomycin (all from Life Technologies, Bleiswijk, the Netherlands), and 1 μM isoproterenol-HCl (Sigma Aldrich, Zwijndrecht, the Netherlands), until approximately 80%–90% confluent.

Differentiation of HBE cells was achieved using air–liquid interface (ALI) culture (further details can be found in the supporting information, S1 File). Alternatively, HBE cell cultures from a homozygous p.Phe508del CF donor (Epithelix Sàrl, Plan-Les-Ouates, Switzerland) were supplied as fully differentiated epithelium grown on permeable supports at ALI and grown in MucilAir medium (Epithelix Sàrl). To ensure that the HBE cells were fully differentiated, the cells were cultured for at least 3 weeks in ALI before the start of treatment.

As opposed to the transfection method used for CFPAC-1 cells, due to difficulties with transfection of eluforsen into differentiated HBE cells (consistent with previous reports [27,28]), continued exposure of the cells to a medium containing Cy5-labeled eluforsen, without transfection reagent, was employed. This technique resulted in a gradual gymnotic uptake of oligonucleotide over time. Cultures were treated with either eluforsen, fluorescent Cy5-labeled eluforsen, or scrambled control oligonucleotide by adding the test compound to the culture medium at final concentrations ranging from 1 nM to 30 μM. This method of administration to HBE cultures was used for both the Ussing chamber experiments and the microscopic analysis of eluforsen uptake.

### In vitro analysis of eluforsen uptake

In order to assess uptake of eluforsen by HBE cells, eluforsen was labeled with Cy5 and administered to primary HBE cultures. HBE cultures were treated from 2 to 28 days with fluorescently labeled eluforsen. After treatment, the cultures were fixed with 4% paraformaldehyde in phosphate-buffered saline (PBS; Life Technologies) and permeabilised with 0.1% Triton X-100 (Promega, Leiden, the Netherlands) in PBS. Next, the cultures were blocked with PBS + 5% bovine serum albumin (Sigma Aldrich), then incubated with Alexa Fluor 488-labeled phalloidin (1:40 in PBS + 5% bovine serum albumin, Life Technologies) to stain for F-actin, and subsequently washed four times with PBS. Hoechst nuclear staining (NucBlue Live ReadyProbes Reagent, Life Technologies) was added to visualize the nucleus. The uptake of Cy5-labeled eluforsen was assessed using an Axio Observer confocal microscope with a Zeiss laser scanning microscope exciter (Zeiss, Sliedrecht, the Netherlands). Pictures were processed using ZEN black software (Zeiss).

### In vitro assessment of eluforsen-mediated restoration of CFTR function: Chloride efflux assay

Chloride efflux from CFPAC-1 cells was assessed using the halide-sensitive fluorescent indicator N-(6-methoxyquinolyl) acetoethyl ester (MQAE; Life Technologies), as has previously been described [29]. CFPAC-1 cells were seeded at a density of 1.5 × 10^4^ or 1.3 × 10^5^ cells/cm^2^ in a black clear-bottom 96-well plate (μClear, 655096, Greiner Bio-One, Alphen aan den Rijn, the Netherlands) and incubated overnight. Next, cells were transfected with 100 nM eluforsen or control oligonucleotide using Lipofectamine 2000 (Life Technologies) (further details can be found in the supporting information, S1 File). The MQAE assay was then performed on these cells. Briefly, cells were incubated in chloride buffer for 15 minutes before being placed in low chloride buffer containing forskolin (Reagent Direct, Encinitas, CA, USA) and 10 μM VX-770 (CFTR potentiator; Selleck Chemicals, Munich, Germany), and the fluorescent signal of MQAE quenching was measured over 2 minutes as a measure of CFTR-mediated chloride efflux (further details can be found in the supporting information, S1 File).

### In vitro assessment of eluforsen-mediated restoration of CFTR function: Ussing chamber assay

In brief, HBE cells were transferred to Ussing chambers with chloride buffer added to the basolateral and the apical side of the cells (further details can be found in the supporting information, S1 File). The difference in CFTR-specific current with eluforsen or scrambled control was measured by adding amiloride to block the predominant sodium channel and testing the difference in short-circuit current (I_sc_) with maximum CFTR activation (by isoproterenol and potentiator [VX-770 or genistein (Sigma Aldrich)]) and CFTR inhibition (by CFTRinh-172 [Sigma Aldrich]).

### Mice

Young adult (12–16 weeks old, males and females, 20–30 g) mice homozygous for the p.Phe508del-CFTR mutation (F508del-CFTR mice), representative of the mutation found in patients with CF, were ordered from Bayer Wuppertal (congenic FVB Cftr^tm1EUR^ [30,31], for saliva secretion assay), bred at the University of Louvain (mixed background 129/FVB Cftr^tm1EUR^ [30,31], for NPD initial experiments), or ordered from The Jackson Laboratory (congenic B6.129S6-Cftr^tm1Kth^/J [32], for further NPD experiments). For biodistribution studies only, C57BL/6 (WT) mice and BALB/c nu/nu (nude) mice (both from Charles River Laboratories, Sulzfeld, Germany) were used. Nude mice were used to allow in vivo imaging of IRDye800-labeled eluforsen, as mouse hairs interfere with fluorescent imaging. For all animal studies, treatment groups were divided over several study cohorts to ensure that measurements were performed throughout the day. Other than equal proportioning of mice of different ages and gender to each treatment group, all animal studies were randomized and analysis of data was blinded. Sample sizes were estimated based on in vitro data, or limited by the available number of animals. Mice were group-housed under specific pathogen-free conditions with reversed light–dark cycle and cage enrichment according to recommendations of the Federation of European Laboratory Animal Science Associations. F508del-CFTR mice were fed with specialized high-protein chow (SM AB diets Breeding, Ssniff Spezialdiäten GmbH, Soest, Germany) to prevent intestinal obstruction. Water and food were supplied ad libitum. Animal experiments were approved by the local Ethics Committee and conformed to the European Community regulations (CEE n° 86/609). The local Ethics Committee was the local animal welfare body of Innoser Laboratories BV (Leiden, the Netherlands) and the project license was granted by the Dutch Central Authority for Scientific Procedures on Animals (CDD).

### Lung administration of eluforsen

For salivary secretion experiments, FVB Cftr^tmi1EUR^ mice (female n = 9, male n = 10) were dosed with eluforsen via oro-tracheal (OT) administration to effectively mimic inhalation, every other day (EOD) up to six doses [33]. Saline was chosen as the in vivo control (female n = 9, male n = 8) due to the unknown safety profile of a scrambled oligonucleotide, and also to correlate with placebo-controlled human clinical trials in which saline would act as the placebo. Mice were anesthetized with a 2.5% isoflurane/2% oxygen and air mixture for at least 3 minutes. The mice were then hung from their front teeth on a plastic line of the mouse intubation platform (model MIP, Penn-Century, Wyndmoor, PA, USA). The nose was blocked using one finger, to prevent nasal breathing, and the tongue was fixed in a protruded position using forceps. A total of 50 μL of formulated eluforsen in saline (5 mg/mL), or saline alone, was administered into the throat using a micropipette. The nose was kept blocked for an additional 20 seconds to allow the test item to be taken up by the lung.

### In vivo imaging and post-mortem detection of eluforsen biodistribution

For in vivo imaging, nude mice were sedated with 2%–3% isoflurane/air mixture and imaged with the Pearl Impulse Small Animal Imaging System (LI-COR Biosciences, Leusden, The Netherlands) at 0 and 15 minutes; 1, 3, 6, 24, 48, 72, and 96 hours; and 7 days after OT dosing of IRDye800-labeled eluforsen (n = 3), IRDye800 dye (n = 1), and unlabeled eluforsen (n = 1). Images were acquired at 800 nm. The fluorescent signal was digitized and electronically displayed as a pseudo-color overlay on a gray-scale white light image of the animal. The data were analyzed using Pearl Impulse Software, version 2.0 (LI-COR Biosciences). For post-mortem detection, nude mice were killed 7 days after OT administration by CO_2_ asphyxiation. Blood was drawn and organs (lung, kidney, trachea, heart, liver, spleen, pancreas, duodenum, and ilium) were removed, snap frozen, and stored at –80°C. Fluorescent signal of IRDye800 in whole organs was detected using an ODYSSEY CLx imaging system (LI-COR Biosciences) and images were acquired.

### Preparation of mouse lung tissue for confocal microscopy detection of Cy5-labeled eluforsen

WT mice (n = 3 per time point; six time points) were killed under anesthesia (2%–3% isoflurane/air mixture) by intra-cardiac puncture. Lungs were filled via the trachea with 0.7 mL 4% paraformaldehyde and incubated overnight. After washing with ethanol and xylol, the tissues were embedded in paraffin overnight. Next, 10 μm sections were cut and deparaffinized. Finally, a drop of 4’,6-diamidino-2-phenylindole (Vector Labs, Amsterdam, the Netherlands) was added to stain the nuclei. Slides were analyzed on a laser scanning microscope observer with Zen Black software (Zeiss).

### Hybridization high-performance liquid chromatography for detection of Cy5

In short, complete lungs of WT mice (n = 3 per time point; six time points) were disrupted using a freezer mill, and lysates prepared with an ultrasonic stick in the presence of tissue and cell lysis solution containing proteinase K (Epicentre, Madison, WI, USA). Cy5-labeled eluforsen was detected using anion-exchange high-performance liquid chromatography (HPLC) with fluorescent detection. A specific fluorescently labeled probe (Atto425-labeled peptide nucleic acid probe, Panagene, Daejeon, Korea) homologous to eluforsen was used to capture eluforsen in serum or tissue lysates, and absolute concentrations were determined by quantitative HPLC measurements using a Shimadzu Fluorescence Detector 20Axs (Shimadzu Corporation, Kyoto, Japan; λexcitation 437 nm; λemission 483 nm).

### In situ hybridization for detection of eluforsen in tissue

The RNAscope 2.0 FFPE Detection Kit (Advanced Cell Diagnostics, Hayward, CA, USA), using customized optimized detection probes complementary to eluforsen, was used to visualize eluforsen in lung tissue.

### In vivo assessment of eluforsen-mediated restoration of CFTR function: Saliva secretion assay in F508del-CFTR mice

Total CFTR-mediated saliva secretion was assessed before treatment and after OT administration of one to six doses of eluforsen (10 mg/kg) or saline. The saliva secretion assay was performed in eluforsen- or saline-treated FVB Cftr^tmi1EUR^ F508del-CFTR mice according to a modified version of the method developed by Best et al. [34]. Briefly, mice were anesthetized by isoflurane, then received a subcutaneous injection with atropine sulfate (50 μL, 1 mM in saline, Sigma Aldrich) in the left cheek. A cotton bud was used to absorb any remaining saliva from the left and right cheeks. At time 0, a mixture (100 μL) of atropine sulfate (1 mM) and isoproterenol (100 μM, Sigma Aldrich) in saline was injected subcutaneously in the left cheek to induce CFTR-mediated saliva production. Subsequently, every 3 minutes for the next 30 minutes, dry, pre-weighed filter paper was positioned in the cheek of the mouse to absorb saliva. The amount of CFTR-mediated saliva secreted was determined by weighing these 10 filter papers and subtracting the dry weight from the total, and then corrected for body weight. Mice were dosed with eluforsen (10 mg/kg; n = 19) or saline (n = 17) by OT administration EOD. The saliva secretion assay was performed 1–7 days pre-treatment (baseline), and 24 hours after receiving one, two, four, and six doses of eluforsen.

### In vivo assessment of eluforsen-mediated restoration of CFTR function: NPD in F508del-CFTR mice

Initial NPD measurements were recorded in fully anesthetized eluforsen-treated F508del-CFTR (FVB/129 Cftr^tm1EUR^) mice (n = 18) or untreated WT littermates (n = 6). Further NPD analyses were carried out in B6.129S6-Cftr^tm1Kth^/J mice treated with eluforsen (n = 10), scrambled control (n = 10), and saline (n = 9). Mice were age-matched per treatment group and an equal ratio of male and female mice were used for these studies. Measurements were carried out as previously described [35]. The baseline (pre-treatment) NPD was measured using isotonic saline Ringer’s solution until a stable value was obtained. Subsequently, a series of solutions were sequentially perfused to assess the nasal ion flow:

1. Amiloride was added to the isotonic saline Ringer’s solution (NaCl [140 mM], CaCl_2_.2H_2_O [2.0 mM], MgCl_2_.6H_2_O [1.0 mM], KCl [6.0], d-Glucose [10 mM], HEPES [10 mM]) to assess the transepithelial potential (V_TE_) _amiloride_.
2. A chloride-free buffer (NaC_6_H_11_O_7_ [140 mM], C_12_H_22_CaO_14_ [6 mM], MgCl_2_.6H_2_O [1.0 mM], C_6_H_11_KO_7_ [6 mM], d-Glucose [10 mM], HEPES [10 mM]) containing amiloride (0.1 mM) was added to assess the V_TE zero Cl_-.
3. A chloride-free buffer (same composition as above) containing amiloride (0.1 mM) and forskolin (10 μM) was added to assess the V_TE forskolin_.

The total CFTR-mediated Cl^−^ conductance was defined as the total change in V_TE_ (ΔV_TE total-Cl¯_) after sequential perfusion with the zero Cl^−^ and forskolin-containing buffers relative to the potential difference after perfusion with the ringer solution containing amiloride. In initial NPD experiments, mice received eluforsen (2 μL, 40 μg/dose) EOD via intranasal (IN) administration in the left nostril using a micropipette. Six doses were administered to ensure sufficient dosing was achieved in the limited available sample. The post-treatment NPD measurement was then recorded 48 hours after the last dose of eluforsen. In the further NPD experiments, mice also received six IN doses of test item EOD (eluforsen, scrambled control, or saline [five administrations at 60-second intervals to a total of 10 μL, 40 μg/dose]). This experiment consisted of two baseline NPD measurements at days 1 and 7, and a post-treatment NPD measurement at day 21 (24 hours after the last dose).

### Statistical analysis

Data and statistics were analyzed using Graphpad Prism 6 and 7 (Graphpad Software, Inc., San Diego, CA, USA). All data were normally distributed. For in vitro experiments and initial NPD experiments, Student’s t-test was used for comparisons between two groups; comparisons involving more than two groups were made using one-way analysis of variance using the appropriate post hoc tests, as detailed in the figure legends. The repeated measures data from the saliva secretion assay were analyzed using analysis of covariance (ANCOVA) and the general linear model function in SAS (SAS, version 9) with the effect of baseline (pre-treatment measurement) and repeated measures as covariates. For further NPD experiments, the effect of treatment with eluforsen on ΔV_TE total-Cl¯_ was compared with the effect of treatment with scrambled control or saline, while taking into account the baseline (pre-treatment) ΔV_TE total-Cl¯_ values. For this purpose, ANCOVA was again used to compare treatment effects at post-treatment NPD by only taking NPD baseline 2 (at day 7) into account as a covariate using the general linear model in SAS (SAS, version 9). Differences were considered statistically significant at p < 0.05.

## Results

### Eluforsen restores CFTR activity in vitro as measured by chloride efflux

**Fig 1:**
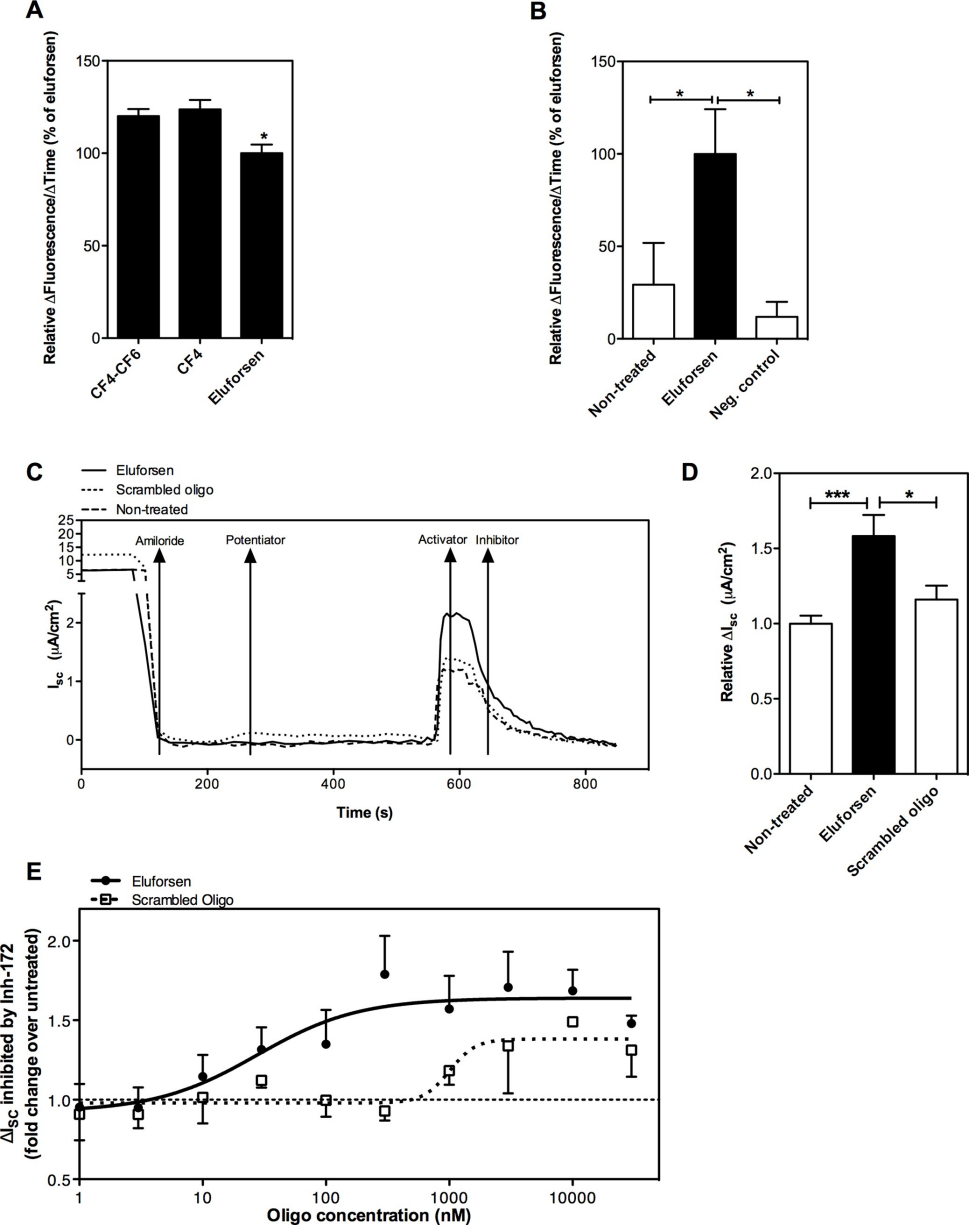
Eluforsen increases chloride efflux in CFPAC-1 cells and restores CFTR function in p.Phe508del CF HBE cells. (A) CFPAC-1 cells transfected with CF4–CF6 duplex or CF4 alone exhibited a slightly higher increase in fluorescence compared with eluforsen-transfected cells (*p < 0.05, n = 4). Mean of values relative to eluforsen ± SEM are shown; differences between treatments were compared by one-way ANOVA with Tukey post hoc test. (B) CFPAC-1 cells transfected with eluforsen exhibited a significantly higher chloride efflux rate (as monitored by MQAE quenching) than untreated cells or negative control oligonucleotide-transfected cells (*p < 0.05, n = 10). Mean of values relative to eluforsen ± SEM are shown; differences between treatments were compared by one-way ANOVA with Tukey post hoc test. (C) Representative I_sc_ traces of isoproterenol-stimulated HBE cultures that were untreated, treated with eluforsen, or treated with scrambled oligonucleotide. (D) CFTR-specific I_sc_ was significantly increased upon eluforsen treatment compared with untreated HBE cultures (***p = 0.0007, n = 20 for untreated, n = 22 for eluforsen) and scrambled control-treated cultures (*p = 0.01, n = 24). Bars show mean ± SEM; differences between treatments were compared by one-way ANOVA with Tukey post hoc test. (E) Dose–response curves of HBE cells treated with eluforsen and scrambled control. The maximum effect of eluforsen is reached at a concentration of 300 nM, with an EC_50_ of 28 ± 2.63 nM. The scrambled control-treated cultures also showed a modest response to treatment at a significantly higher concentration (EC_50_ 1,002 ± 1.42 nM, p < 0.01, n = 70 for eluforsen and n = 40 for scrambled control). Dots show mean ± SEM; the differences between the EC_50_s of the dose responses was compared by Student’s t-test.

The in vitro effect of eluforsen on chloride efflux was assessed using MQAE, a fluorescent molecule that is quenched by chloride ions and is therefore used as a measure of intracellular chloride concentration. The effect of eluforsen was compared with that of the CF4–CF6 duplex or CF4 alone. Eluforsen has the same sequence and 2’-O-methyl modifications as CF4, and phosphorothioate modifications were introduced to promote uptake by cells in vivo (see eluforsen uptake below). Forskolin is used to stimulate CFTR-mediated chloride efflux. Using the MQAE chloride efflux assay, it was shown that the level of chloride efflux in CFPAC-1 cells was similar after treatment with the CF4–CF6 duplex or CF4 alone (Fig 1A), confirming that CF4 is the active component of the duplex and sufficient for restoration of CFTR-mediated chloride efflux. Eluforsen-induced increases in chloride efflux rates were slightly smaller than those observed for CF4 and CF4–CF6 (eluforsen: 100% versus CF4–CF6: 120% versus CF4: 124%), suggesting a small impact of the phosphorothioate modifications on the function of eluforsen. Nonetheless, eluforsen was shown to significantly increase chloride efflux compared with scrambled control and untreated cells (Fig 1B), indicating that eluforsen also restores CFTR-mediated chloride efflux.

Functional repair of CFTR by eluforsen was further assessed by measuring chloride conductance in HBE cells grown in ALI to mimic the situation in the lung. Compounds, such as eluforsen, were added to the medium to enable gymnotic uptake by the cells. I_sc_ responses of HBE cells incubated with eluforsen or scrambled control were measured using the Ussing chamber assay. In this assay, I_sc_ is an indicator of net ion transport taking place across the epithelium. Primary HBE cultures were chosen to maximize clinical relevance. The data showed that incubation of HBE cells grown in ALI culture with eluforsen resulted in an increase in I_sc_ over time, with a maximum effect observed after 14 days of incubation (S1A Fig); an incubation period of 14 days was therefore used in subsequent functional assessments of eluforsen in HBE cultures. Eluforsen treatment significantly improved I_sc_compared with untreated cells and scrambled control-treated cells (Fig 1C and 1D). The increase in I_sc_ after eluforsen treatment was abolished by CFTRinh-172, indicating that the restorative effect of eluforsen in primary HBE ALI cultures was mediated by CFTR. This effect was observed in primary cells from multiple donors, and was observed both in the presence and absence of a potentiator (S1B Fig).

To assess the dose–response effect of eluforsen, HBE cultures were treated with either eluforsen or scrambled control at concentrations ranging from 1 nM to 30 μM. The resulting dose–response curve shows that eluforsen starts to be effective above 3 nM, with a maximum effect at a concentration of 300 nM, and an EC_50_ of 28 ± 2.63 nM (Fig 1E). In contrast, the scrambled control showed no effect until a concentration of 1,000 nM, with an EC_50_ of 1,002 ± 1.42 nM (p < 0.01 for EC_50_ versus eluforsen).

### Eluforsen demonstrated intracellular presence following orotracheal administration

**Fig 2:**
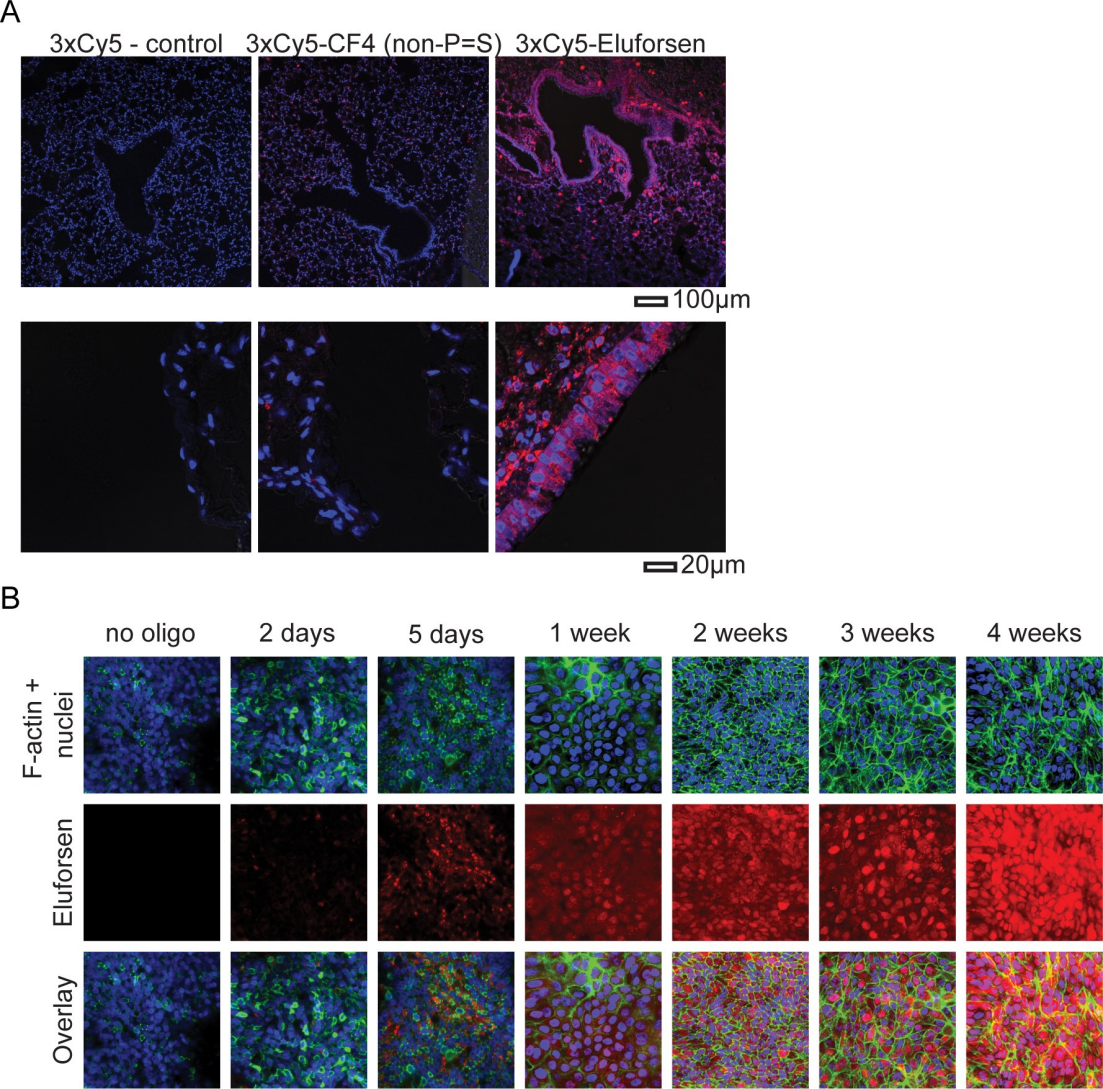
Cy5-labeled eluforsen is visualized in the airway epithelial layer after OT administration. (A) Representative confocal images of lung paraffin sections of wild-type mice that received daily OT administration of Cy5 (control), Cy5-labeled CF4, or Cy5-labeled eluforsen (10 mg/kg; equimolar dose) for 3 consecutive days and were killed on day 4. Nuclei were stained with DAPI (shown in blue). (B) Representative confocal images of HBE cultures treated with Cy5-labeled eluforsen (shown in red) stained for the nucleus (Hoechst, blue) and F-actin (Alexa Fluor 488-phalloidin, green).

Assessment of lung sections from WT mice using confocal microscopy indicated that the Cy5 signal of Cy5-labeled eluforsen, but not CF4, could be detected in the airway epithelium 4 days after OT administration (Fig 2A). This finding suggests the phosphorothioate modifications improved the cellular uptake of Cy5-labeled eluforsen compared with (non-phosphorothioate-modified) Cy5-labeled CF4. In addition to the in vivo assessment of eluforsen, confocal microscopy of primary homozygous p.Phe508del-CFTR HBE cells incubated with eluforsen in vitro showed that Cy5-labeled eluforsen accumulated over a period of 4 weeks (Fig 2B).

### Biodistribution of eluforsen in vivo

**Fig 3:**
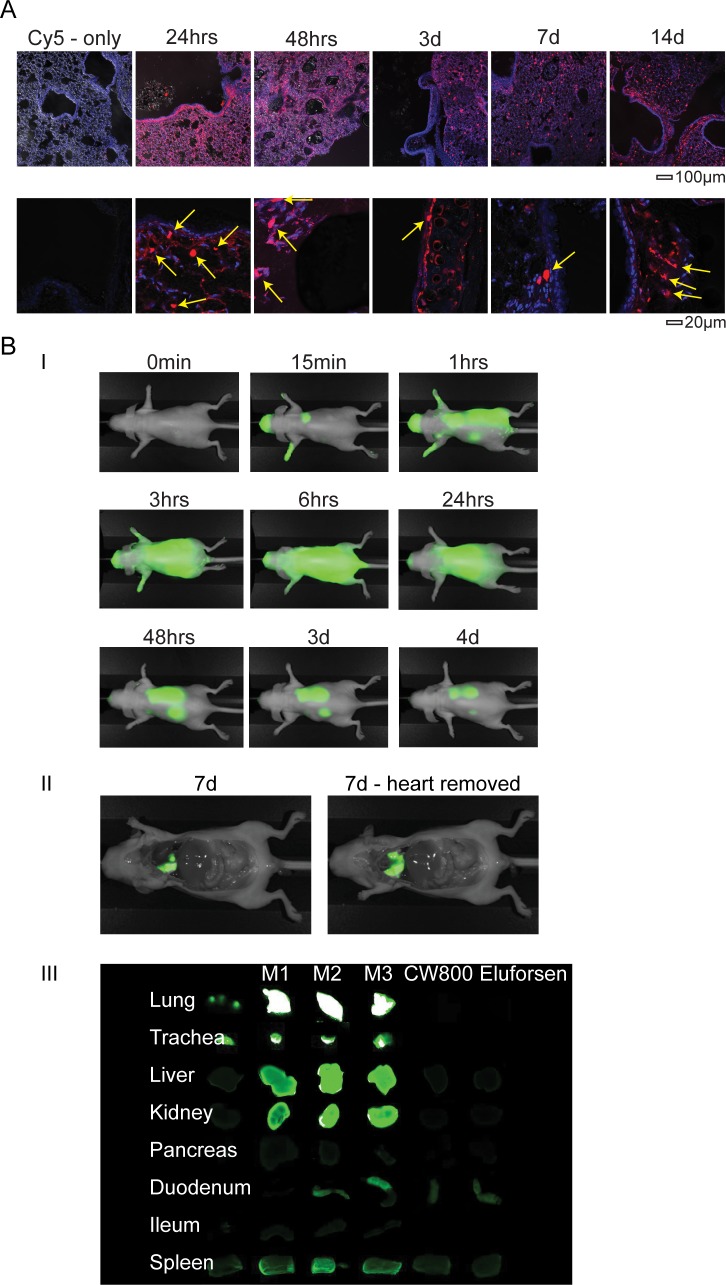
In vivo imaging and post-mortem detection demonstrate systemic exposure after OT administration of labeled eluforsen. (A) Representative confocal images of lung paraffin sections of wild-type (WT) mice following a single OT administration of 10 mg/kg Cy5-labeled eluforsen (shown in red) and killed after 24 hours, 48 hours, 3 days, 7 days, and 14 days. The cell nucleus was stained with DAPI (shown in blue). Macrophages accumulate the Cy5-labeled eluforsen and are visible as highly fluorescent spherical cells (as indicated with the yellow arrows). (B) (i) Representative in vivo images at several time points showing the IRDye800 (CW800) signal from IRDye800-labeled eluforsen in green. Systemic exposure could be detected at 1 hour after administration. (ii) Mice were killed after 7 days and representative in situ images demonstrate a strong IRDye800 signal in the lungs. (iii) High-resolution post-mortem scans of organs from all animals show extrapulmonary distribution of eluforsen. Controls include a mouse that received an equimolar dose or IRDye800 alone (CW800) or unlabeled eluforsen. Mouse M1 shown (see S4 Fig for additional animals).

To assess eluforsen uptake and accumulation in vivo, Cy5-labeled eluforsen was administered OT and Cy5 signal was visualized over time. At 24 hours, Cy5 signal was detected in the epithelial cells lining the airway lumen and in macrophages, and over time the signal decreased. At later time points, the majority of the Cy5 signal was detected in macrophages. HPLC analysis indicated that the Cy5 signal originated primarily from intact Cy5-labeled eluforsen (S2 Fig). In treated WT mice, Cy5-labeled eluforsen could be detected in post-mortem sections up to 14 days after OT administration (Fig 3A). In vivo imaging data of IRDye800-labeled eluforsen in nude mice confirmed uptake by the airway epithelium and subsequent biodistribution to extrapulmonary organs including the liver, kidney, spleen, and gastrointestinal tract (Fig 3B and S4 Fig). No Cy5 could be detected after 24 hours in untreated mice that received Cy5 only. Unlabeled eluforsen was measurable in blood 30 minutes after OT administration in both WT and F508del-CFTR mice (S3 Fig) using hybridization HPLC. Moreover, unlabeled eluforsen was also detected in the liver, kidney, and salivary glands after airway delivery (S3 Fig). In addition visualization of unlabeled eluforsen using in situ hybridization showed that eluforsen is present in the bronchi-epithelium, septa of the alveoli, and macrophages (S3 Fig).

### Eluforsen restores CFTR-mediated saliva secretion in F508del-CFTR mice

**Fig 4:**
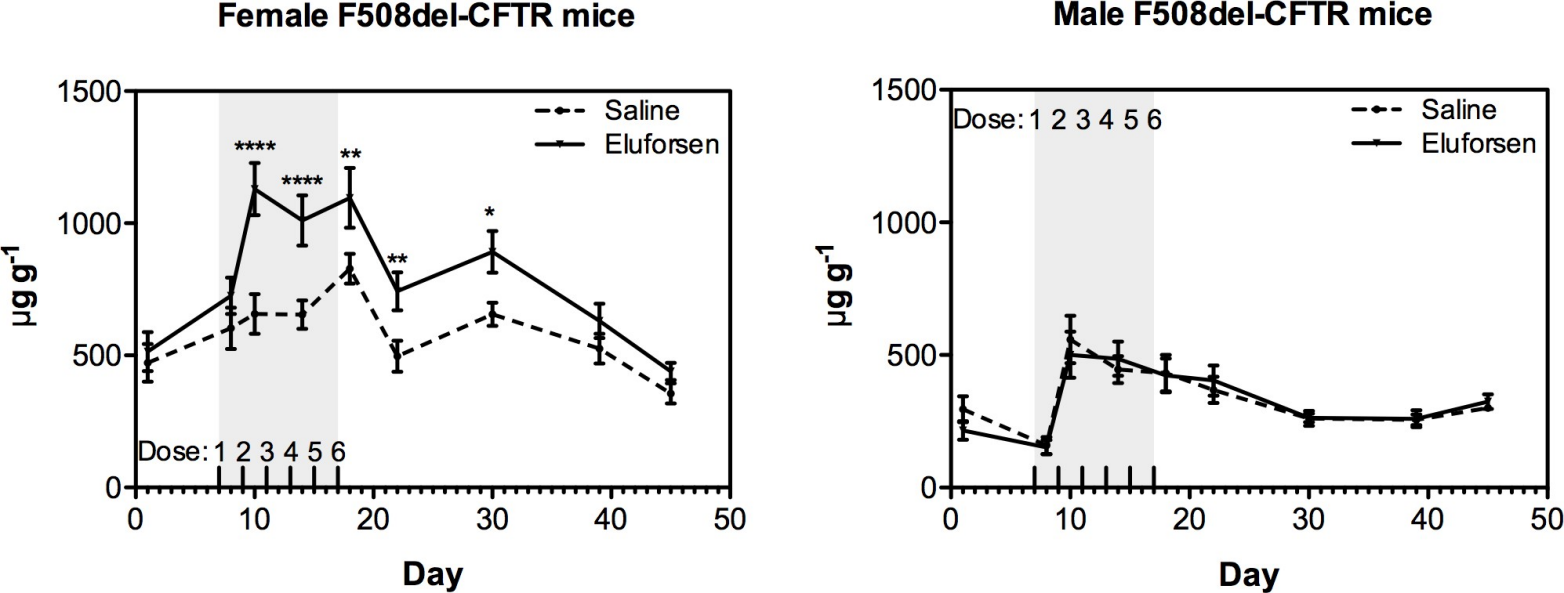
Eluforsen restores CFTR-mediated saliva secretion in female F508del-CFTR mice. CFTR-mediated saliva secretion in eluforsen-treated F508del-CFTR mice before treatment (at day 1) and 24 hours after one (day 8), two (day 10), four (day 14), and six (days 18, 22, 30, 39, 45) OT administrations of saline or eluforsen (10 mg/kg), corrected for body weight (μg saliva/g body weight). The symbols and error bars indicate the mean and SEM. The solid line represents the mean CFTR-mediated saliva secretion corrected for body weight in eluforsen-treated (female n = 9; male n = 10) mice and the dashed line represents the saline-treated (female n = 9; male n = 8) mice. Two to six OT administrations of eluforsen significantly improve CFTR-mediated salivary secretion in female F508del-CFTR mice compared with saline-treated controls. CFTR-mediated saliva secretion gradually returned to pre-treatment levels at day 39 (22 days after the sixth dose), confirming washout of eluforsen. Treatment groups were compared using ANCOVA (general linear model), using the effect of baseline (pre-treatment measurement) and repeated measures as covariates. ****p < 0.0001, **p < 0.01, *p < 0.05.

As in the sweat glands of patients with CF, the salivary glands of F508del-CFTR mice fail to respond to β-adrenergic stimulation [35,36]. Therefore, saliva secretion resulting from β-adrenergic stimulation may be used as a measure of CFTR function. Firstly, uptake of eluforsen by the salivary gland after OT administration was confirmed using hybridization HPLC (S3B Fig). Saliva measurements indicated a significant increase in CFTR-mediated saliva secretion after two doses of eluforsen in female F508del-CFTR mice compared with pre-treatment levels and saline control (p < 0.0001), but no difference was detected in male mice (Fig 4). In female F508del-CFTR mice, two to six doses of eluforsen strongly increased CFTR-mediated saliva secretion. The effect of eluforsen persisted for up to 13 days after dosing, and was not significantly different from saline-treated mice after 22 days (Fig 4). The percent change from baseline saliva secretion as assessed a week prior to treatment is attached in (S6 Fig).

### Eluforsen normalizes NPD in F508del-CFTR mice

**Fig 5:**
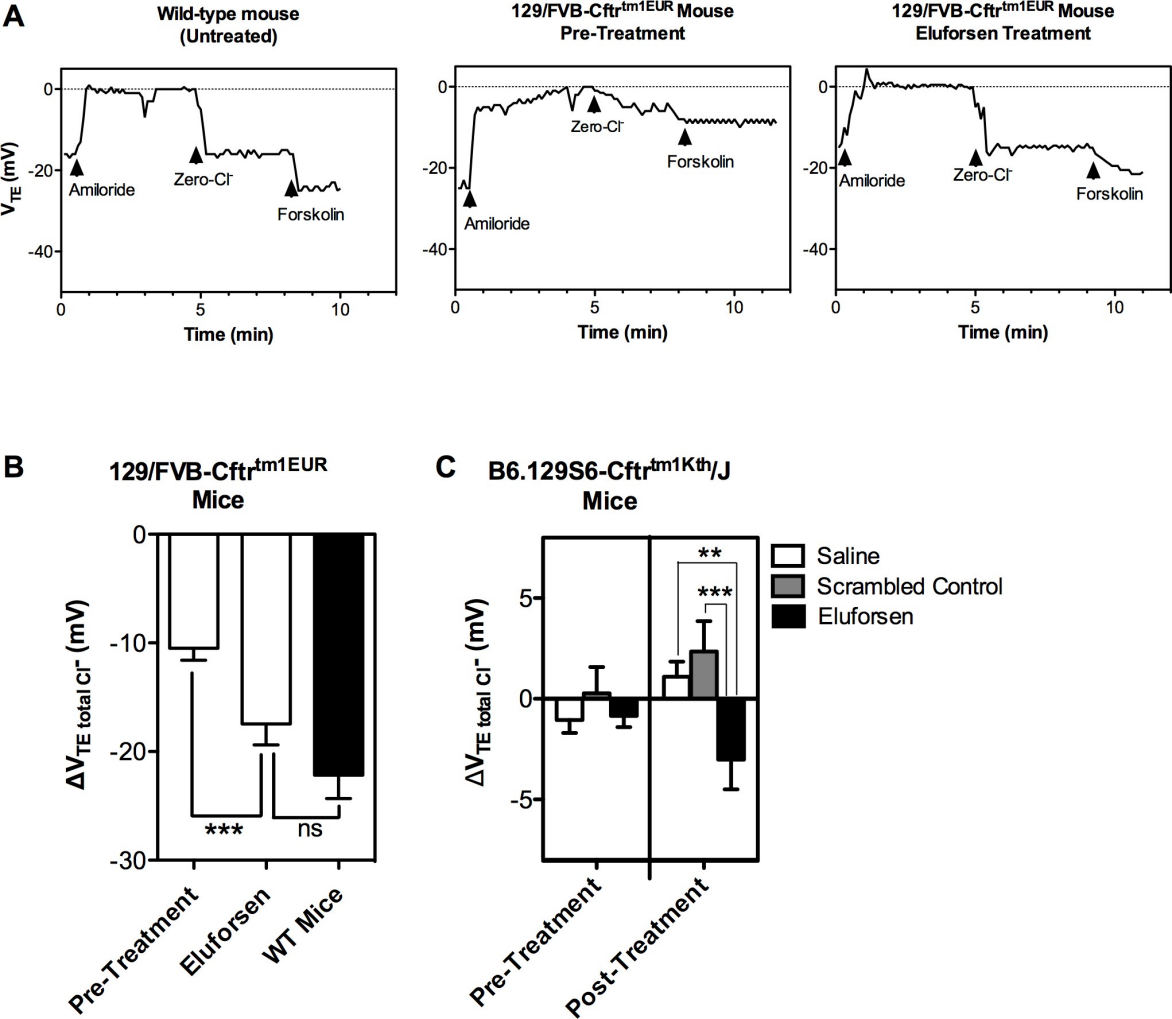
Eluforsen restores NPD in two different strains of F508del-CFTR mice. (A) Representative NPD traces in WT littermates, untreated 129/FVB Cftr^tm1EUR^ mice, and eluforsen-treated 129/FVB Cftr^tm1EUR^ mice. (B) ΔV_TE total-Cl_- obtained from NPD recordings in 129/FVB Cftr^tm1EUR^ mice at pre-treatment and after six intranasal doses (EOD) of 40 μg eluforsen (open bars; n = 18), and in untreated WT littermates (black bars; n = 6). Eluforsen significantly increases CFTR-mediated chloride permeability compared with pre-treatment levels, and to approximately 80% of WT levels. Bars show mean ± SEM. ΔV_TE total-Cl_- values in mice before and after eluforsen treatment were compared by paired t-test (***p = 0.0001); the differences in ΔV_TE total-Cl_- values between eluforsen-treated and WT littermates were compared by unpaired t-test (ns: non-significant). (C) In B6.129S6-Cftr^tm1Kth^/J mice, the ΔV_TE total-Cl¯_ measurements at baseline were similar between treatment groups, but less negative compared with 129/FVB Cftr^tm1EUR^ mice. At 24 hours post-treatment, eluforsen-treated mice (n = 10) had significantly improved ΔV_TE total-Cl¯_ (more negative) compared with both saline (n = 9) and the scrambled control (n = 10). Bars show mean ± SEM. The effect of eluforsen treatment on ΔV_TE total-Cl¯_ values was compared with the ANCOVA control groups using the pre-treatment NPD value (**p = 0.015, ***p = 0.0001).

The bioelectric abnormalities observed in NPD measurements in patients with CF and p.Phe508del mutation and in F508del-CFTR mice are very similar, with both showing a large inhibition of NPD in the presence of amiloride (sodium channel inhibitor), and little or no change in response to zero luminal chloride [37]. Treatment with six IN administrations of eluforsen over two weeks in 129/FVB Cftr^tm1EUR^ mice (n = 18) resulted in significant improvements in the increase in total V_TE_ differences (ΔV_TE_) after perfusion with a zero-chloride buffer containing forskolin (ΔV_TE total-Cl_-; p = 0.0005) (Fig 5A and 5B). This effect was not observed when only three IN administrations over one week were received (n = 5 mice; S5A Fig). The effect of eluforsen on ΔV_TE total-Cl_- diminished 10 days after receiving the last of six IN doses (n = 6 mice; S5B Fig). These data confirm the significant improvement in CFTR function in homozygous F508del-CFTR mice with eluforsen treatment. Due to the relatively high background ΔV_TE total-Cl_- value in 129/FVB Cftr^tm1EUR^ compared with their WT littermates, and preliminary data showing lower background ΔV_TE total-Cl¯_ in B6.129S6-Cftr^tm1Kth^/J mice,^28^ the NPD assay was also carried out in B6.129S6-Cftr^tm1Kth^/J mice. These mice were treated with IN eluforsen as well as saline and scrambled control to further verify the treatment effect. As shown in the 129/FVB Cftr^tm1EUR^ mice, treatment with eluforsen significantly improved the CFTR-mediated Cl¯ conductance compared with saline (p = 0.015) or scrambled controls (p = 0.001) (Fig 5C) in B6.129S6-Cftr^tm1Kth^/J mice. There was no effect of gender observed on the NPD outcome parameters for either mouse strain.

## Discussion

This study shows that eluforsen is a bioavailable RNA oligonucleotide therapy that results in functional CFTR in p.Phe508del-CFTR-mediated CF in in vitro and in vivo models. In two in vitro models using two different assays, eluforsen restores chloride conductance. The first model described in this report show that eluforsen significantly improves CFTR-mediated chloride efflux compared with untreated and control-treated CFPAC-1 cells. Chloride efflux assays are frequently used to verify functional CFTR with pharmacologic treatment [38]. However, CFPAC-1 cells show a very low level of CFTR expression [39–41], and thus may not accurately reflect the level of effect that may be achieved in lung epithelium. Therefore, the effect of eluforsen on CFTR activity in differentiated primary p.Phe508del HBE cell cultures, grown in ALI to mimic the lung epithelium, was also tested. Called the Ussing chamber, this model also allows for measurement of chloride currents, which reflect CFTR activity. While a transfection agent was used to enhance intracellular uptake of eluforsen in CFPAC-1 cells, eluforsen was taken up by p.Phe508del-CFTR HBE cells after continued exposure in the medium. Exposure time to eluforsen correlated with a significant improvement in CFTR activity as measured by increased I_sc_ responses in the Ussing chamber assay of p.Phe508del-CFTR HBE cells compared with scrambled control-treated p.Phe508del-CFTR HBE cells. Although the scrambled control appears to have a small but non-significant effect on I_sc_ at higher concentrations, the EC_50_ of eluforsen is significantly lower than that of the scrambled control. The increased I_sc_ seen with the scrambled oligonucleotide could be attributed to non-sequence-specific effects, for example caused by the phosphorothioate modifications, frequently observed with control oligonucleotides at very high concentrations [42]. Given the origin and expansion of primary HBE cell lines, it is challenging to compare absolute results either from experiment to experiment or from p.Phe508del-CFTR HBE to WT HBE cells. Nonetheless, these two in vitro models demonstrate transmembrane chloride conductance following exposure to eluforsen. In addition, OT administration of eluforsen achieved good uptake and persistence of this oligonucleotide in pulmonary epithelial cells; extrapulmonary distribution of eluforsen was also observed after OT administration.

The murine model of homozygous p.Phe508del-CFTR-mediated CF [30,31] has previously been described as excellently suited for studying the pharmacology of therapeutic agents intended to restore CFTR function [34,43–45]. Moreover, the RNA at the Phe508del site in this murine model is 100% homologous to the human RNA at this same site [30–32]. As such, this model was considered suitable to test eluforsen, an antisense oligonucleotide synthesized following the human Phe508del site RNA sequence.

The respiratory route of administration was chosen as a way to target the large surface area of the airway epithelium where CFTR is densely expressed, as well as to avoid the first pass effect in the liver that is well recognized with antisense oligonucleotide therapies administered systemically. There are barriers to uptake of unmodified oligonucleotides or small double-stranded RNA sequences by the airway epithelium, and subsequent distribution to other organs [46]. However, the results presented here demonstrate rapid uptake by airway epithelium of both fluorescently labeled and unlabeled eluforsen after OT administration, and is distributed via systemic circulation to extrapulmonary organs (mostly the liver and kidney, but also detected in the gastrointestinal tract and salivary glands). Importantly, OT administration simulates inhalation, which is the intended clinical route of eluforsen administration. Moreover, Cy5-labeled eluforsen levels remain stable in the lungs for at least 14 days after OT administration. This prolonged in vivo stability of eluforsen in the target tissue is important considering the expected short half-life of the CFTR protein and mRNA in epithelial cells [47–49]. Importantly, these data show that the lung is also a portal of entry for systemic delivery of eluforsen to other extrapulmonary organs that are affected by CF. The results of the present study suggest that extrapulmonary distribution of eluforsen to target organs is achieved after OT administration. Uptake by alveolar macrophages is also noted, consistent with their primary function of clearing foreign debris from the lungs.

We also investigated the pharmacodynamic effects of eluforsen in vivo. Clinically relevant assays were used to assess the effect of eluforsen on CFTR function in two different epithelial tissues: the salivary glands and nasal epithelium. The saliva secretion assay is the murine equivalent of the sweat test that is used as a primary or surrogate endpoint in human clinical trials [34,36]. The failure of salivary glands in F508del-CFTR mice to respond to β-adrenergic stimulation corresponds to the failure of sweat glands in patients with CF to secrete fluid in response to β-adrenergic stimulation [50]. OT delivery of two to six doses of eluforsen restored CFTR-mediated saliva secretion in a dose-dependent manner in female p F508del-CFTR mice. This effect was observed up to 13 days after the last dose, and was not significantly different from pre-treatment after 22 days. No effect of eluforsen on CFTR-mediated salivary production was observed in male F508del-CFTR mice. However, this is not surprising considering previously reported differences in the responses of male and female mice in the saliva secretion assay, suggested to be related to morphologic, chemical/hormonal, and physiologic variations between male and female salivary glands [36]. In addition, it was noted that in the saliva secretion assay study, the saline-treated female mice also showed increased saliva secretion over time. This increased secretion is attributed to the effect of the anesthetics (isoflurane and multiple administrations of atropine and isoproterenol within relatively short time intervals). Since the saliva secretion levels in female eluforsen-treated mice increased much earlier than levels in saline-treated mice, the early improvement is considered to be an eluforsen-specific effect.

NPD is highly dependent on functioning CFTR. Since the early 1980s, measurement of NPD has been used extensively for the diagnosis of CF as the measurements are sensitively and specifically different when there is no functioning CFTR [51]. In addition to use as a diagnostic tool specific to patients with CF, NPD is also currently being used as a surrogate marker of CFTR activity in several clinical trials [22–24,51]. Using this measure of chloride conductance in mutant F508del-CFTR (129/FVB Cftr^tm1EUR^) mice and B6.129S6-Cftr^tm1Kth^/J mice compared with their respective WT mice, it was found that IN administration of eluforsen restores chloride conductance in F508del-CFTR mice. This effect was dose-dependent; a minimum of six doses was needed to achieve functional correction. After six IN doses, a washout of eluforsen was observed with NPD returning to pre-treatment levels after 10 days. This mirrors the findings of a phenotypic reversal of p.Phe508del previously reported by Zamecnik et al. in cultured cells [21], and is in agreement with the reversibility of increased saliva production observed in the saliva secretion assay. These data further support the hypothesis of eluforsen-mediated functional restoration of p.Phe508del-CFTR.

In summary, the results presented in this report demonstrate that eluforsen, a single-stranded, chemically modified RNA oligonucleotide, improved CFTR function in two vitro models and in two in vivo models using mice with the p.Phe508del-CFTR genotype. These encouraging preclinical data served as the foundation for two human clinical trials of eluforsen in patients with p.Phe508del-CFTR-mediated CF (NCT02564354 and NCT02532764). For one of these two studies, NPD was measured in patients before and after intranasal administration of eluforsen and showed restoration of CFTR function, further confirming the murine NPD data described here [52]. Moreover, data from a phase 1b dose escalation study demonstrated an improvement in CFQ-R RSS, a relevant measure of clinical benefit in CF patients, after eluforsen treatment [53].

## Supporting information

S1 File Supporting materials and methods. (DOCX)

S1 Fig Uptake of eluforsen by HBE cells over time results in improved CFTR function. (A) I_sc_ measurements of HBE cultures showed improved chloride permeation over time, with optimal response after 2 weeks of treatment (n = 3). The decrease in I_sc_ after 4 weeks of treatment may indicate deterioration in cell viability, corresponding to the irregular staining of the HBE cells seen by confocal microscopy in Fig 1B. (B) I_sc_ measurements of HBE cultures from two additional donors showing similar increases in current in cells treated with eluforsen, but not in cells treated with scrambled control. This was observed both with and without concomitant potentiator (genistein) treatment (n = ≥4. Bars show mean ± SEM).
(TIFF)

S2 Fig Cy5 signal detected in lung tissue corresponds to Cy5 bound to eluforsen. Total Cy5 signal was detected using hybridization HPLC. Percentages of Cy5-labeled eluforsen (intact), Cy5-labeled metabolites of eluforsen (truncated eluforsen with Cy5 label), and free Cy5 as part of the total Cy5 signal in lung tissue at 24 hours, 7 days, and 14 days after OT administration of Cy5-labeled eluforsen. The exact molecular entities of the truncated eluforsen with Cy5 label could not be identified with the current method, but were expected to consist of eluforsen without 1 to 3 nucleotides from the ‘3 end. The bar represents the mean percentage of each analyte, with n = 2 mice per time point. The majority (~75%) of the Cy5 signal is from intact Cy5-labeled eluforsen 24 hours and 7 and 14 days after OT administration. The percentage of Cy5 corresponding to truncated eluforsen was increased at 14 days after OT administration. At all time points measured, the amount of free Cy5 was very low (< 5%), indicating that the Cy5 signal detected in the lung corresponds to eluforsen-bound Cy5.
(TIFF)

S3 Fig Biodistribution of eluforsen in WT mice after OT administration. WT mice received a single OT administration of eluforsen (10 mg/kg), which resulted in rapid absorption by the lung, systemic exposure to blood (A), and rapid biodistribution to the liver, kidney, and salivary gland. (B) Hybridization HPLC shows that eluforsen concentration in all organs stabilizes within the first 24 hours, and remains stable for a week. The maximum concentration in serum is reached 2–4 hours after OT administration, and remains stable near lower detection levels after 24 hours (n = 3 mice per time point). (C) In situ hybridization shows that eluforsen (brown, left side) was detected in the bronchi-epithelium, septa of the alveoli, and macrophages (as indicated with arrows) of WT mice 24 hours after a single OT administration of eluforsen. No eluforsen was detected in saline-treated WT mice (right side).
(TIF)

S4 Fig In vivo imaging of IRDye800-labeled eluforsen in nude mice. Nude mice (M2 and M3) were dosed via OT administration with IRDye800-labeled eluforsen, and absorption by the airway epithelium and biodistribution to extrapulmonary organs were assessed by in vivo imaging and post-mortem detection. Several time points after OT administration show the IRDye800 signal in green. Systemic exposure could be detected at 1 hour after administration. Mice were killed after 7 days, and representative in situ images demonstrate a strong IRDye800 signal in the lungs. The signal from IRDye800 (CW800) alone disappeared 6 hours after dosing, suggesting a different biodistribution profile. No signal was detected in the mouse treated with unlabeled eluforsen.
(TIF)

S5 Fig Effect of eluforsen on CFTR-mediated chloride permeability in 129/FVB Cftr^tm1EUR^ mice. (A) Eluforsen increased CFTR-mediated chloride permeability in 129/FVB Cftr^tm1EUR^ mice after six (n = 18; in 14 days), but not three (n = 5; in 7 days) intranasal doses (40 μg/dose) EOD as shown by the ΔV_TE total-Cl_- parameters. Mean ± SEM shown. ΔV_TE total-Cl_- values in F508del-CFTR mice before and after eluforsen treatment were compared by paired t-test (***p = 0.0005). ΔV_TE total-Cl_- values between eluforsen-treated F508del-CFTR mice and WT littermates were compared by unpaired t-test (ns). (B) Washout effect on ΔV_TE total-Cl_- in post-treatment (n = 18), 10 days post-treatment (n = 6), and 17 days post-treatment (n = 2) in 129/FVB Cftr^tm1EUR^ mice, showing return to pre-treatment levels within 10 days. Bars show mean ± SEM. ΔV_TE_ parameters before and after eluforsen treatment were compared by paired t-test (***p = 0.0005).
(TIFF)

S6 Fig Eluforsen restores CFTR-mediated saliva secretion in female F508del-CFTR mice. The percent change from baseline (day 1) CFTR-mediated saliva secretion in eluforsen-treated F508del-CFTR mice after 24 hours and after one (day 8), two (day 10), four (day 14), and six (days 18, 22, 30, 39, 45) OT administrations of saline or eluforsen (10 mg/kg). The symbols and error bars indicate the mean and SEM. The solid line represents the mean percentage (female n = 9; male n = 10) mice and the dashed line represents the saline-treated (female n = 9; male n = 8) mice. Treatment groups were compared using ANCOVA (general linear model), using the effect of baseline (pre-treatment measurement) and repeated measures as covariates. ****p < 0.0001, **p < 0.01, *p < 0.05.
(TIF)

## Abbreviations:

ALI: Air-liquid interface
ANCOVA: Analysis of covariance
CF: Cystic fibrosis
CFPAC-1: Cystic fibrosis pancreatic adenocarcinoma-1
CFTR: Cystic fibrosis transmembrane conductance regulator
EOD: Every other day
HBE: Human bronchial epithelial
HPLC: High-performance liquid chromatography
IN: intranasal
MQAE: *N*-(6-methoxyquinolyl) acetoethyl ester
NPD: Nasal potential difference
nt: Nucleotide
OT: Oro-tracheal
PBS: Phospate-buffered saline
p.Phe508del: Phenylalanine at position 508
WT: Wild-type
