# Supplementary material for: Evaluation of eluforsen, a novel RNA oligonucleotide for restoration of CFTR function in in vitro and murine models of p.Phe508del cystic fibrosis
Source: PLoS One. 2019 Jun 28;14(6):e0219182. doi: 10.1371/journal.pone.0219182 (PMC6599119; doi:10.1371/journal.pone.0219182)
Supplement: S1 File — (DOCX) [file pone.0219182.s001.docx]

**Supporting materials and methods**

**ALI culture**

Human bronchial epithelial (HBE) cells were seeded on 6.5 mm Transwell permeable supports (Corning, Amsterdam, the Netherlands). These supports were coated with 300 µL phosphate-buffered saline (PBS) containing 30 µg/mL PureCol (CellSystems Biotechnologie Vertrieb GmbH, Troisdorf, Germany), 10 µg/mL fibronectin (Sigma Aldrich), and 10 µg/mL bovine serum albumin (BSA) for 2 hours at 37°C and 5% CO_2_. Next, the HBE cells were seeded at a density of 4.5 × 10^5^ cells/cm^2^ in differentiation medium consisting of 1:1 bronchial epithelial cell growth medium (BEGM; Lonza, Castleford, UK) and Dulbecco’s modified Eagle’s medium (DMEM; Life Technologies), BEGM SingleQuot Kit Supplements & Growth Factors (Lonza, Breda, the Netherlands), 1% penicillin/streptomycin, 1.5 µg/mL BSA, and 15 ng/mL retinoic acid (Sigma Aldrich). The medium was refreshed three times per week, and once the cells had formed a fully confluent monolayer, the medium was removed from the apical side to establish an air–liquid interface (ALI). The cultures were maintained for at least 3 weeks in ALI before the start of treatment.

**Transfection of CFPAC-1 cells with eluforsen**

Eluforsen oligonucleotide and Lipofectamine 2000 were mixed at a 1:5 v/v ratio in Opti-MEM medium (Life Technologies), incubated at room temperature for 5 minutes, then added to the culture medium. After 6 hours, the transfection medium was replaced by culture medium and the cells were cultured for an additional 24–72 hours before being used for the *N*-(6-methoxyquinolyl) acetoethyl ester (MQAE) assay.

**MQAE assay**

CF pancreatic adenocarcinoma (CFPAC-1) cells were loaded for 6 hours with 10 mM MQAE in culture medium. Next, the cells were washed twice with chloride buffer (130 mM NaCl, 5 mM KCl, 1.2 mM CaCl_2_, 1 mM MgCl_2_, 10 mM glucose, and 20 mM [4-(2-hydroxyethyl)-1-piperazineethanesulfonic acid; HEPES]) and incubated for 15 minutes with chloride buffer. The buffer was then removed completely and replaced by low chloride buffers (130 mM NaNO_3_, 5 mM KCl, 1.2 mM CaCl_2_, 1 mM MgCl_2_, 10 mM glucose, and 20 mM HEPES) containing 10 µM forskolin and 10 µM VX-770 (ivacaftor [CFTR potentiator]). Fluorescence signal at 460 nm was measured every 30 seconds using a FluoDia T70 plate reader (Photon Technology International, Longjumeau, France) and the rate of increase in fluorescence signal over the first 2 minutes was used as a measure of eluforsen-mediated restoration of CFTR-mediated chloride efflux.

**Ussing chamber**

HBE cells grown on Transwell permeable supports were transferred to Ussing chambers (P2300, Physiological Instruments, San Diego, CA, USA) mounted in a heat block kept at 37°C, and chloride buffer (135 mM NaCl, 2.4 mM K_2_HPO_4_, 0.6 mM KH_2_PO_4_, 1.2 mM CaCl_2_, 1.2 mM MgCl_2_, 10 mM glucose, and 10 mM HEPES) was added to the basolateral and apical side of the cells. The cultures were left to equilibrate for 10 minutes prior to measuring the I_sc_. Transepithelial voltage (V_TE_) was clamped to 0 mV, and I_sc_ was measured using a VCC MC6 Voltage/Current Clamp (Physiologic Instruments, San Diego, CA, USA) and digitally recorded using Acquire and Analyze software (version 2.3; Physiologic Instruments). The CFTR-specific current was measured by sequential addition of:

1. 30 µM amiloride (Sigma Aldrich) to the apical side (block the predominant sodium channel ENaC);

2. 5 µM VX-770 or 30 μM Genistein (CFTR potentiation);

3. 100 µM isoproterenol (CFTR activation) (Sigma Aldrich); and

4. 30 µM CFTRinh-172.

The difference between the maximum I_sc_ after CFTR stimulation and the I_sc_ after specific CFTR inhibition was calculated as a measure of CFTR activity.
