# Supplementary figures and images for: Evaluation of eluforsen, a novel RNA oligonucleotide for restoration of CFTR function in in vitro and murine models of p.Phe508del cystic fibrosis
Source: PLoS One. 2019 Jun 28;14(6):e0219182. doi: 10.1371/journal.pone.0219182 (PMC6599119; doi:10.1371/journal.pone.0219182)

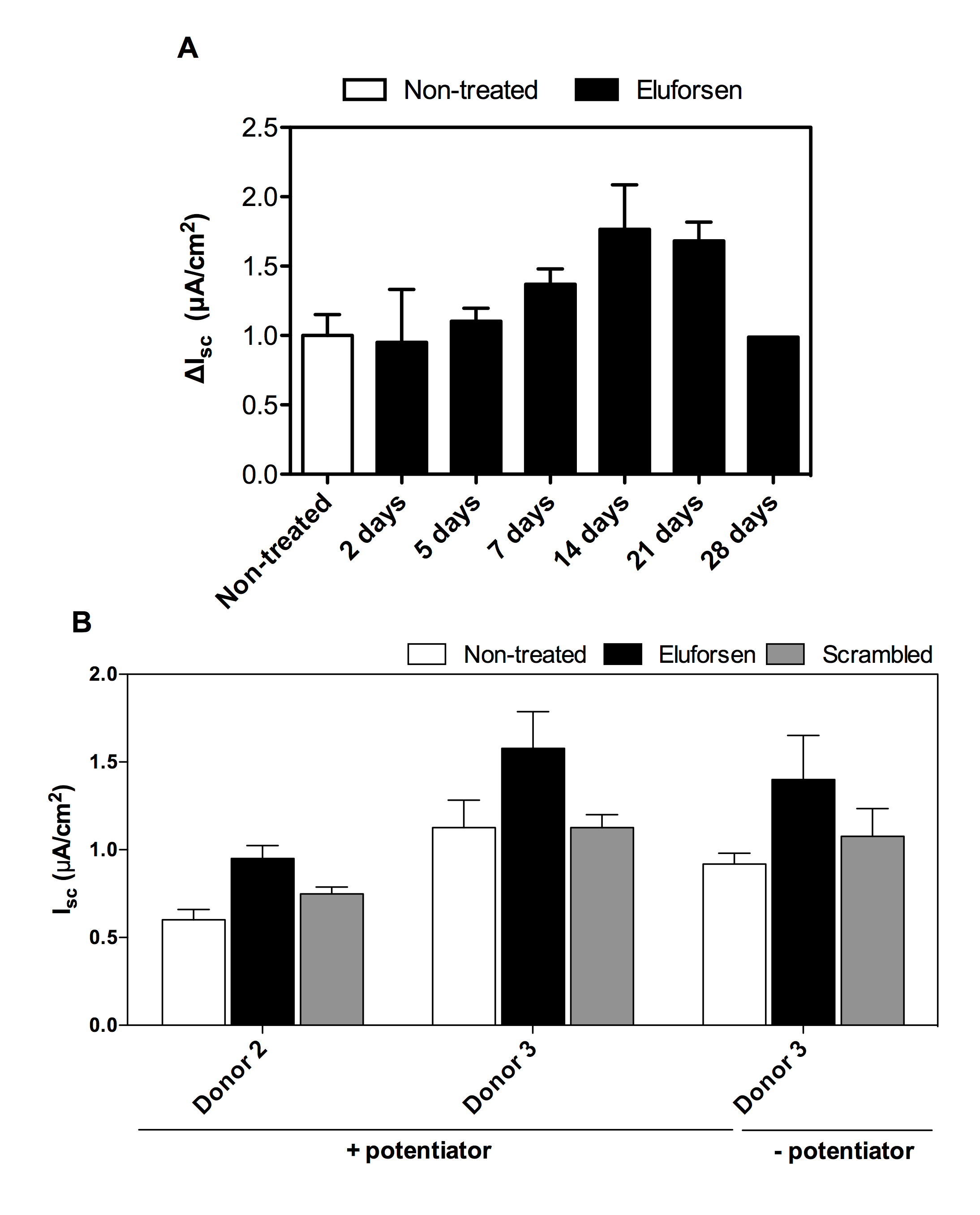

Supplement: S1 Fig — (A) Isc measurements of HBE cultures showed improved chloride permeation over time, with optimal response after 2 weeks of treatment (n = 3). The decrease in Isc after 4 weeks of treatment may indicate deterioration in cell viability, corresponding to the irregular staining of the HBE cells seen by confocal microscopy in Fig 1B. (B) Isc measurements of HBE cultures from two additional donors showing similar increases in current in cells treated with eluforsen, but not in cells treated with scrambled control. This was observed both with and without concomitant potentiator (genistein) treatment (n = ≥4. Bars show mean ± SEM). (TIFF) [file pone.0219182.s002.tiff]

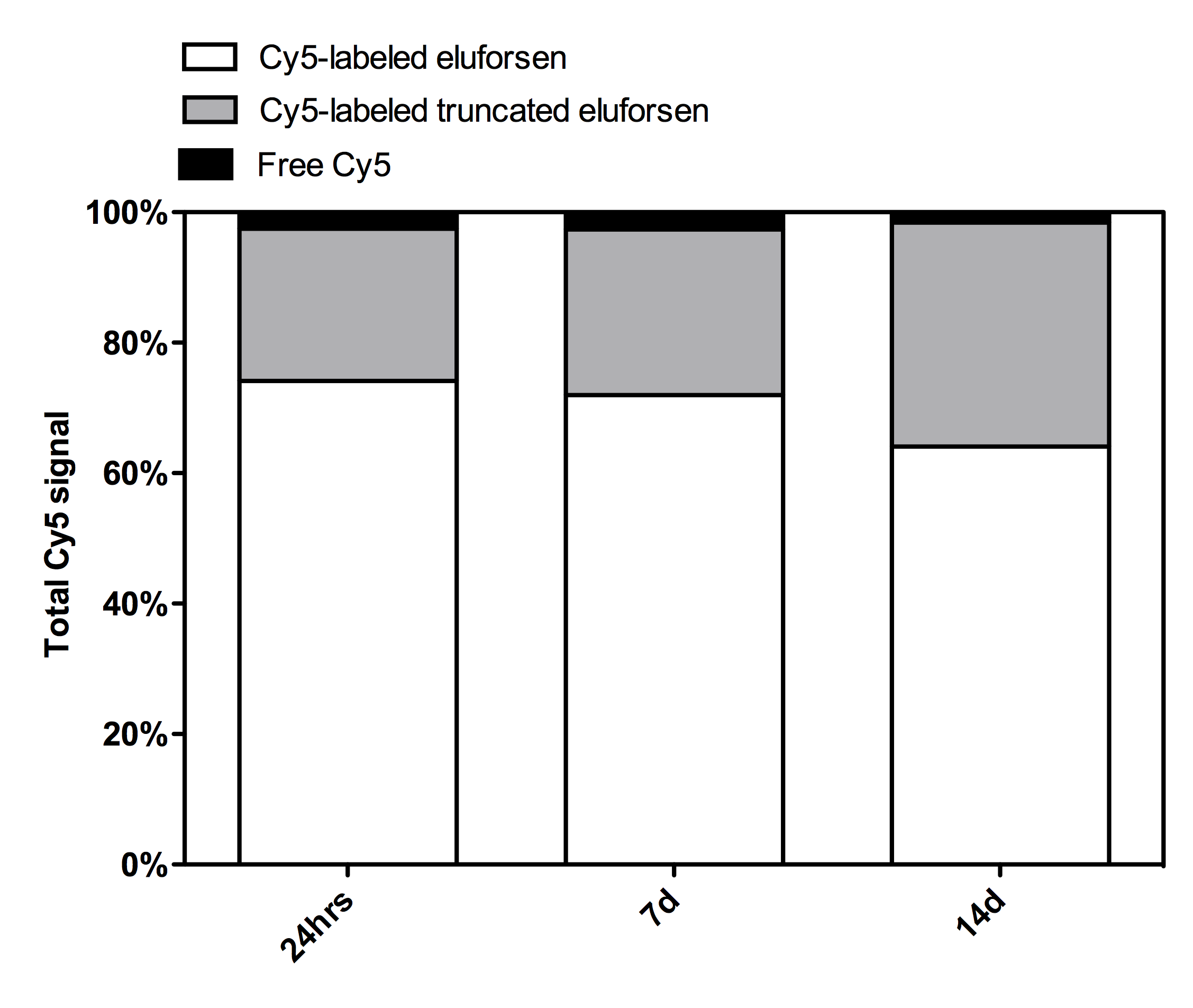

Supplement: S2 Fig — Total Cy5 signal was detected using hybridization HPLC. Percentages of Cy5-labeled eluforsen (intact), Cy5-labeled metabolites of eluforsen (truncated eluforsen with Cy5 label), and free Cy5 as part of the total Cy5 signal in lung tissue at 24 hours, 7 days, and 14 days after OT administration of Cy5-labeled eluforsen. The exact molecular entities of the truncated eluforsen with Cy5 label could not be identified with the current method, but were expected to consist of eluforsen without 1 to 3 nucleotides from the ‘3 end. The bar represents the mean percentage of each analyte, with n = 2 mice per time point. The majority (~75%) of the Cy5 signal is from intact Cy5-labeled eluforsen 24 hours and 7 and 14 days after OT administration. The percentage of Cy5 corresponding to truncated eluforsen was increased at 14 days after OT administration. At all time points measured, the amount of free Cy5 was very low (< 5%), indicating that the Cy5 signal detected in the lung corresponds to eluforsen-bound Cy5. (TIFF) [file pone.0219182.s003.tiff]

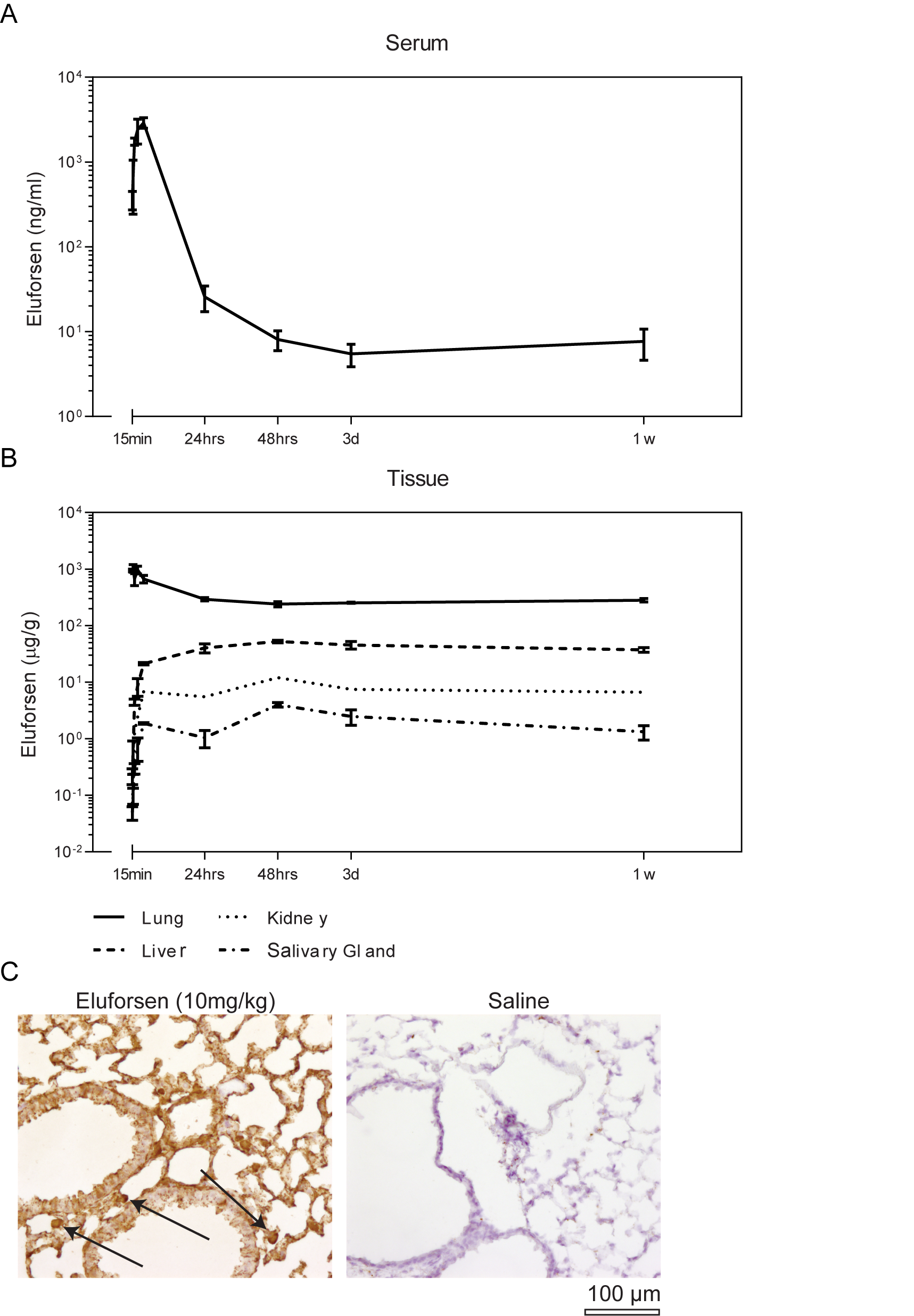

Supplement: S3 Fig — WT mice received a single OT administration of eluforsen (10 mg/kg), which resulted in rapid absorption by the lung, systemic exposure to blood (A), and rapid biodistribution to the liver, kidney, and salivary gland. (B) Hybridization HPLC shows that eluforsen concentration in all organs stabilizes within the first 24 hours, and remains stable for a week. The maximum concentration in serum is reached 2–4 hours after OT administration, and remains stable near lower detection levels after 24 hours (n = 3 mice per time point). (C) In situ hybridization shows that eluforsen (brown, left side) was detected in the bronchi-epithelium, septa of the alveoli, and macrophages (as indicated with arrows) of WT mice 24 hours after a single OT administration of eluforsen. No eluforsen was detected in saline-treated WT mice (right side). (TIF) [file pone.0219182.s004.tif]

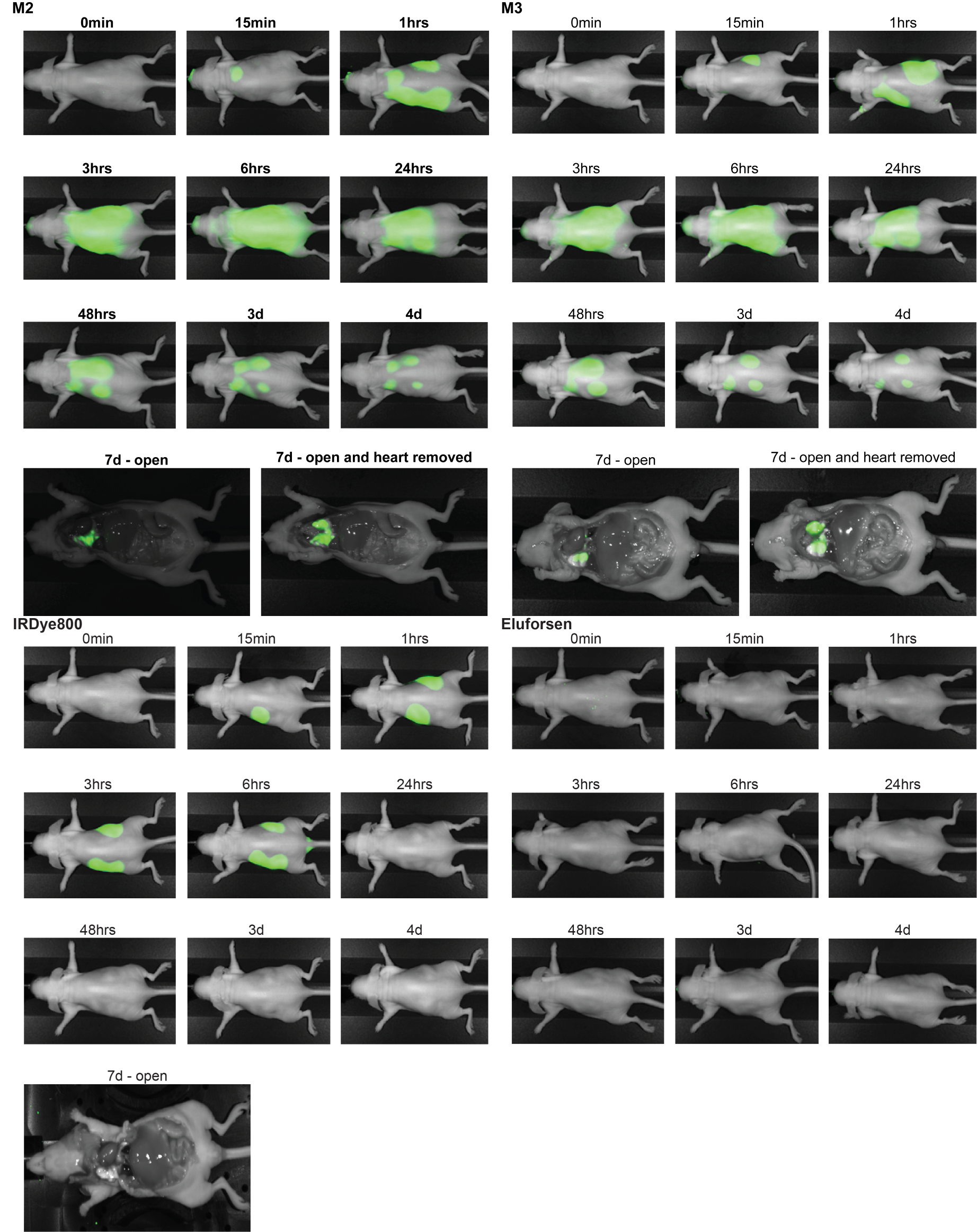

Supplement: S4 Fig — Nude mice (M2 and M3) were dosed via OT administration with IRDye800-labeled eluforsen, and absorption by the airway epithelium and biodistribution to extrapulmonary organs were assessed by in vivo imaging and post-mortem detection. Several time points after OT administration show the IRDye800 signal in green. Systemic exposure could be detected at 1 hour after administration. Mice were killed after 7 days, and representative in situ images demonstrate a strong IRDye800 signal in the lungs. The signal from IRDye800 (CW800) alone disappeared 6 hours after dosing, suggesting a different biodistribution profile. No signal was detected in the mouse treated with unlabeled eluforsen. (TIF) [file pone.0219182.s005.tif]

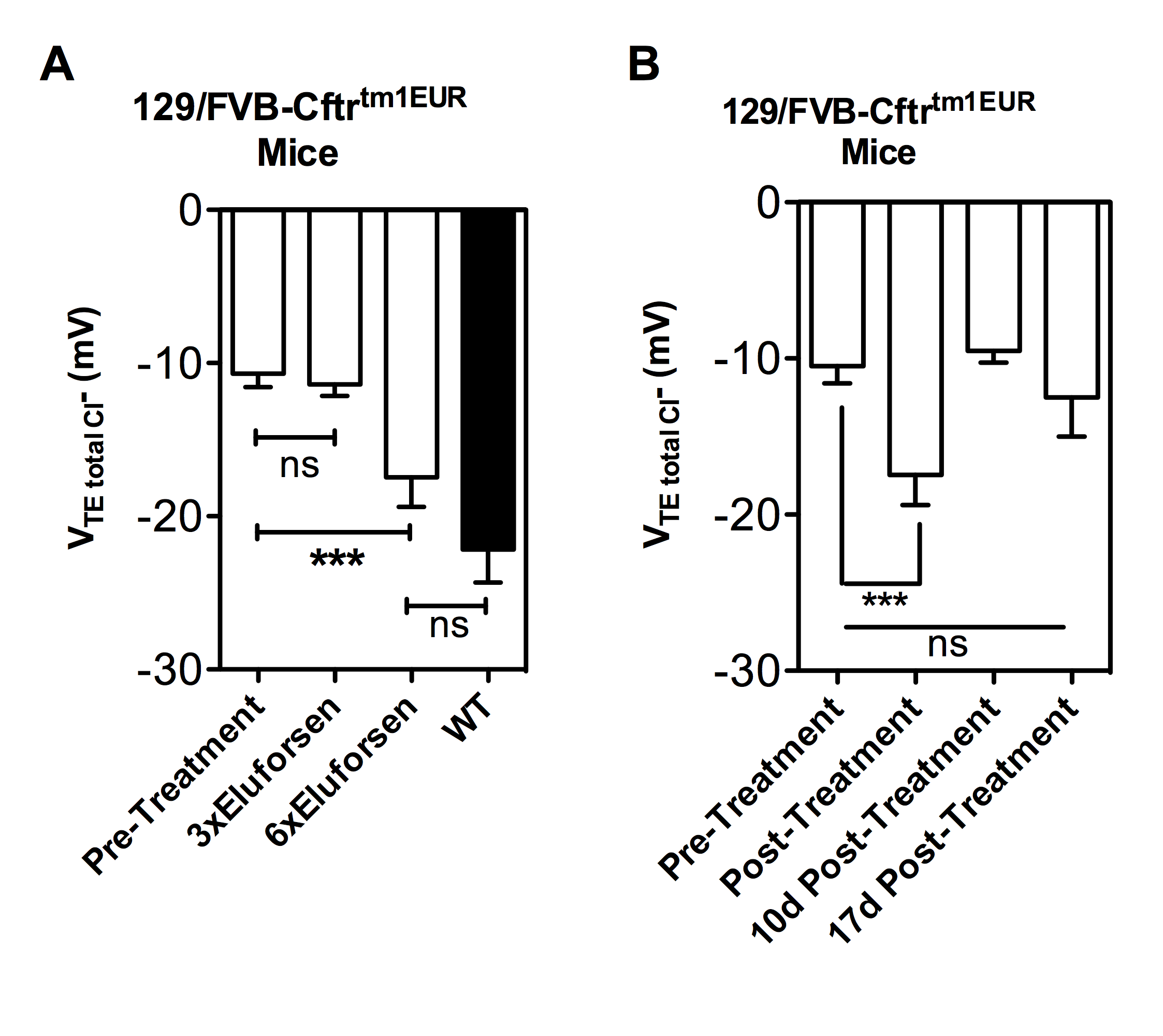

Supplement: S5 Fig — (A) Eluforsen increased CFTR-mediated chloride permeability in 129/FVB Cftrtm1EUR mice after six (n = 18; in 14 days), but not three (n = 5; in 7 days) intranasal doses (40 μg/dose) EOD as shown by the ΔVTE total-Cl- parameters. Mean ± SEM shown. ΔVTE total-Cl- values in F508del-CFTR mice before and after eluforsen treatment were compared by paired t-test (***p = 0.0005). ΔVTE total-Cl- values between eluforsen-treated F508del-CFTR mice and WT littermates were compared by unpaired t-test (ns). (B) Washout effect on ΔVTE total-Cl- in post-treatment (n = 18), 10 days post-treatment (n = 6), and 17 days post-treatment (n = 2) in 129/FVB Cftrtm1EUR mice, showing return to pre-treatment levels within 10 days. Bars show mean ± SEM. ΔVTE parameters before and after eluforsen treatment were compared by paired t-test (***p = 0.0005). (TIFF) [file pone.0219182.s006.tiff]

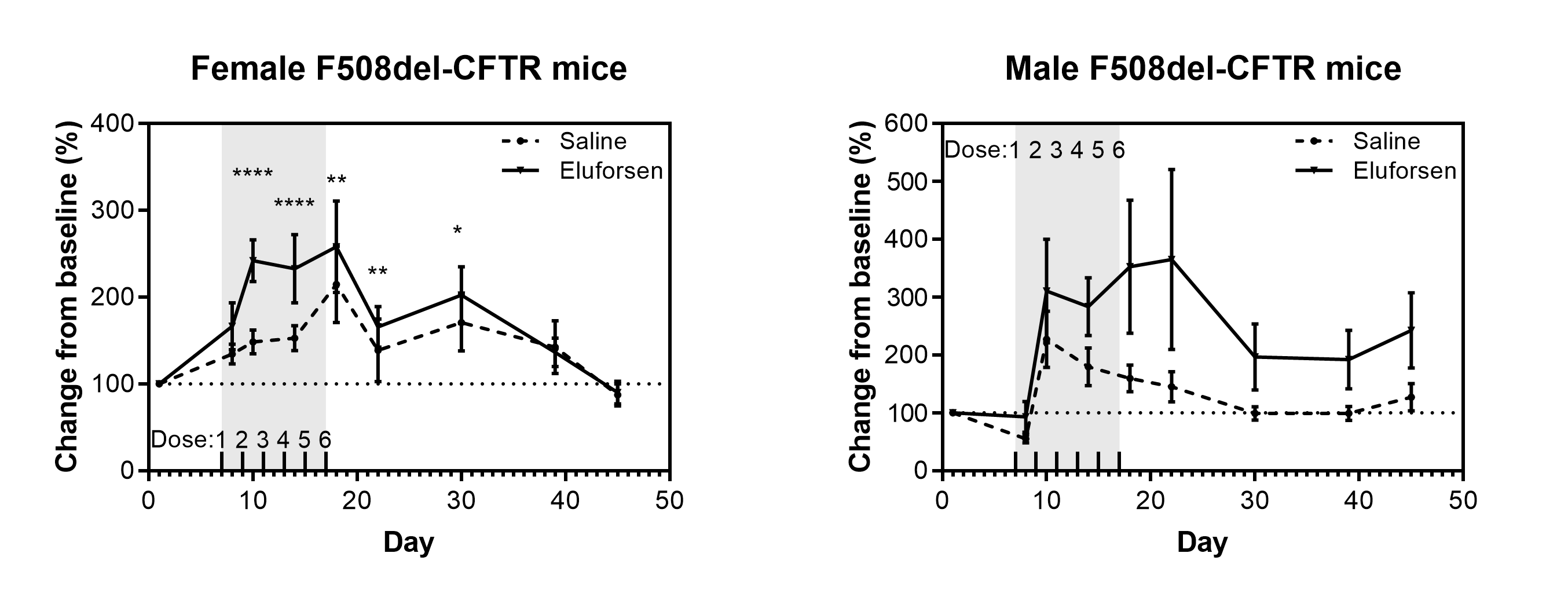

Supplement: S6 Fig — The percent change from baseline (day 1) CFTR-mediated saliva secretion in eluforsen-treated F508del-CFTR mice after 24 hours and after one (day 8), two (day 10), four (day 14), and six (days 18, 22, 30, 39, 45) OT administrations of saline or eluforsen (10 mg/kg). The symbols and error bars indicate the mean and SEM. The solid line represents the mean percentage (female n = 9; male n = 10) mice and the dashed line represents the saline-treated (female n = 9; male n = 8) mice. Treatment groups were compared using ANCOVA (general linear model), using the effect of baseline (pre-treatment measurement) and repeated measures as covariates. ****p < 0.0001, **p < 0.01, *p < 0.05. (TIF) [file pone.0219182.s007.tif]
